# Modeling the Time-Course of Responses for the Border Ownership Selectivity Based on the Integration of Feedforward Signals and Visual Cortical Interactions

**DOI:** 10.3389/fpsyg.2016.02084

**Published:** 2017-01-20

**Authors:** Nobuhiko Wagatsuma, Ko Sakai

**Affiliations:** ^1^School of Science and Engineering, Tokyo Denki UniversitySaitama, Japan; ^2^Department of Computer Science, University of TsukubaTsukuba, Japan

**Keywords:** figure-ground segregation, border ownership, neural dynamics, computational model, visual perception, attention, early vision, surrounding suppression/facilitation

## Abstract

Border ownership (BO) indicates which side of a contour owns a border, and it plays a fundamental role in figure-ground segregation. The majority of neurons in V2 and V4 areas of monkeys exhibit BO selectivity. A physiological work reported that the responses of BO-selective cells show a rapid transition when a presented square is flipped along its classical receptive field (CRF) so that the opposite BO is presented, whereas the transition is significantly slower when a square with a clear BO is replaced by an ambiguous edge, e.g., when the square is enlarged greatly. The rapid transition seemed to reflect the influence of feedforward processing on BO selectivity. Herein, we investigated the role of feedforward signals and cortical interactions for time-courses in BO-selective cells by modeling a visual cortical network comprising V1, V2, and posterior parietal (PP) modules. In our computational model, the recurrent pathways among these modules gradually established the visual progress and the BO assignments. Feedforward inputs mainly determined the activities of these modules. Surrounding suppression/facilitation of early-level areas modulates the activities of V2 cells to provide BO signals. Weak feedback signals from the PP module enhanced the contrast gain extracted in V1, which underlies the attentional modulation of BO signals. Model simulations exhibited time-courses depending on the BO ambiguity, which were caused by the integration delay of V1 and V2 cells and the local inhibition therein given the difference in input stimulus. However, our model did not fully explain the characteristics of crucially slow transition: the responses of BO-selective physiological cells indicated the persistent activation several times longer than that of our model after the replacement with the ambiguous edge. Furthermore, the time-course of BO-selective model cells replicated the attentional modulation of response time in human psychophysical experiments. These attentional modulations for time-courses were induced by selective enhancement of early-level features due to interactions between V1 and PP. Our proposed model suggests fundamental roles of surrounding suppression/facilitation based on feedforward inputs as well as the interactions between early and parietal visual areas with respect to the ambiguity dependence of the neural dynamics in intermediate-level vision.

## Introduction

Neural mechanisms for separating a figural object from the background is a fundamental process necessary for scene perception and object recognition. A number of psychological studies have clarified the phenomenological characteristics and importance of figure-ground perception from a variety of aspects such as perceptual grouping and organization, attentional selection, three-dimensional (3D) representation, and perception of illusory contours (Sporns et al., 1991; He and Nakayama, 1992; Kimchi et al., 2007; Matsukura et al., 2007; Russell et al., 2014). Physiological studies have shown that figure-ground segregation starts in early- to intermediate-level visual cortical areas with neurons selective to the direction of figure (DOF; Lamme, 1995; Sajda and Finkel, 1995). Zhou et al. (2000) reported that a number of neurons in V2 and V4 show the selectivity to border ownership (BO): the responses of the cells depended on which side of the contour owned the border. Although neural mechanisms underlying the BO selectivity are currently under investigation (Qiu et al., 2007; Dong et al., 2008; Martin and von der Heydt, 2015), computational studies have provided insightful evidence from a number of aspects (e.g., Zhaoping, 2005; Supér et al., 2010). Specifically, spatial characteristics of BO selectivity have been studied extensively from a variety of aspects, including contour groupings, attentional modulation, DOF discrimination, and the representations of shape (Oh and Choe, 2007; Wagatsuma et al., 2008; Mihalas et al., 2011; Grossberg, 2016).

Temporal characteristics of BO-selective cells appear to be crucial for further understanding the essence of neural mechanisms underlying the perception of figure-ground segregation. Physiological studies have reported short latencies (50–100 ms) for the discrimination of BO, and 50–180 ms for the attentional modulation of the BO signals (Zhou et al., 2000; Qiu et al., 2007). Computational analyses have suggested the crucial roles of feedforward and rapid feedback processing. Computational models (Sakai and Nishimura, 2006; Supér et al., 2010) have suggested that the feedforward signals mediating the surrounding contrasts around the classical receptive field (CRF) of neurons in V1 and V2 underlie the selectivity of BO. Craft et al. (2007) proposed a grouping structure in V4 that would induce the BO selectivity in V2 via feedback. These feedforward and feedback models showed the characteristic short latency for the BO discrimination and is in good agreement with the electrophysiology. Another model based on the local interactions among V2 neurons gradually establishes the figure-ground segregation and the representation of 3D shape with longer latencies, which is inconsistent with the electrophysiology (Zhaoping, 2005). A more interesting observation in the time-course of BO-selective cell responses is its dependence on the ambiguity of figure-ground cues (O'Herron and von der Heydt, 2009). The responses of the cells exhibit a rapid transition when a presented square is flipped along its CRF so that the opposite BO is presented, whereas the transition is significantly slower when the square is replaced by an edge with ambiguous BO. This phenomenon appears to be key for clarifying the neural mechanisms underlying the determination of BO. We note that the images projected onto the CRF of BO-selective cells were identical during stimulus presentations (a side of a square or an edge). This leads naturally to our hypothesis that the distinct transitions of the responses depend on the distribution of contrasts surrounding the CRF. Clarification of the mechanisms underlying the time-course of BO-selective cells will provide crucial insights into the perceptual mechanism of figure-ground segregation.

We have focused on understanding the role of surround modulation in the determination of BO. Our previous computational and psychophysical studies have indicated that the surrounding suppression/facilitation observed in early visual areas (Jones et al., 2001, 2002; Ozeki et al., 2009) plays crucial roles in the responses of BO-selective cells (Sakai and Nishimura, 2006; Sakai et al., 2012). Specifically, we showed that early-level features such as luminance contrast around the CRF are capable of allocating BO in a manner similar to the physiological observations. This mechanism was extended to deal with the algorithms for representing the medial axis that was the precursor of the perception of 2D shape and 3D objects (Hatori and Sakai, 2014; Qiu et al., 2015). Our previous study on a network model based on surround modulation showed that attention applied to early visual processing underlies the modulation of the activities of BO-selective cells which evokes the alternation of figural objects in ambiguous images (Wagatsuma et al., 2008, 2013a). The model showed good agreements with human perception in the attentional modulation of the response magnitude. The model included the dynamics of the model cells that activate the mutual interactions between the visual areas in order to realize the alternation of attentional effects. However, neither temporal characteristics nor time-courses of BO-selective cells have been examined. It is crucial to investigate whether the surround modulation reproduces the temporal characteristics of BO-selective cell. Specifically, the examination of the time-course of BO-selective cells and its dependence on the ambiguity of DOF would provide crucial information for understanding the BO selectivity. We expect that the integration of feedforward signals via surrounding suppression/facilitation is responsible for the rapid and slow transitions of BO signals when distinct and ambiguous DOF are presented, respectively. Because the surround/feedforward mechanism change directly the cellular responses based on the spatial distribution of stimulus contrast. The surround/feedforward mechanism may provide the latency sensitive to the transition. Other mechanisms such as feedback projections and local intra-cortical interactions may evoke longer and similar latencies in the transitions of BO signals. If the surrounding suppression/facilitation in early visual areas is a key factor for the activities of BO-selective cells, our proposed network models exhibit the time-course of the responses by BO-selective cells depending on the ambiguity of the DOF. This investigation also implies whether the cortical interactions between early and parietal visual areas for attentional modulation in addition to the surround modulation in early-level areas are consistent with the characteristics of response time in human psychophysical experiments. To our knowledge, this is the first computational work for studying the figure-ground-cue-dependent time-course of BO-selective cells.

In the present study, we investigated the roles of the integration of feedforward signals via surrounding suppression/facilitation and the cortical interactions between early and parietal visual areas for the time-course of the responses of BO-selective cells in the extrastriate cortex through simulation of a computational model that comprised of V1, V2, and posterior parietal (PP) modules (Wagatsuma et al., 2008). In this model, the PP module represented the dorsal pathway and was designed to represent an object's location based on the luminance contrast. Top-down spatial attention increased the responses by the PP to enhance the representation of the attended location (Rolls and Deco, 2002; Deco and Lee, 2004). Feedback from the PP module altered the contrast gain in the V1 module, which, in turn, modulated the activities of BO-selective model cells in the V2 module as the responses of these cells were determined by the surrounding suppression/facilitation based on early-level features extracted in the V1 (Sakai and Nishimura, 2006; Sakai et al., 2012). Herein, we performed the simulations of our model with various visual inputs, which corresponded to physiological and psychophysical experiments. The stimuli corresponding to physiological experiments were mainly used to test the influences of the feedforward inputs for the time-course of BO-selective cell responses. In our proposed model, the time-course of the responses by BO-selective cells was dependent on the ambiguity of DOF. This ambiguity dependence was caused by the integration delay of feedforward inputs and the local inhibition due to the difference in input stimulus. However, our model did not fully explain the persistent responses over the next second as shown in BO-selective physiological cells when the square was replaced by the ambiguous edge. In contrast to these physiological stimuli, the psychophysical stimuli were used to investigate the roles of the V1-PP interactions mediating the attentional modulations in the model. The model reproduced the perceptual modulation induced by spatial attention (Posner, 1980). Our current model also predicted an attention-dependent time-course for BO-selective cells in which a short latency was observed when attention was directed to the BO side but a longer latency for no attention and a much longer latency when attention was directed to the opposite side. The interactions between PP and V1 modules resulted in these attentional modulations for temporal characteristics of the responses by BO-selective cells. These results suggest that, at least in part, the surrounding suppression/facilitation based on early-level features, as well as cortical interactions between early and parietal visual areas, play important roles in the neural dynamics depending on the ambiguity of DOF and in the attentional modulation of human perception in figure-ground segregation.

## Materials and methods

### Model architecture

In a previous computational study (Sakai and Nishimura, 2006), it was proposed that the cortical mechanism underlying BO coding is involved with the surrounding early-level features such as luminance contrast and surrounding suppression/facilitation observed in the striate area (Jones et al., 2001, 2002; Ozeki et al., 2009). Although this model was rather abstract in that the responses of BO-selective model cells were determined solely by the balance of early-level features, it successfully reproduced the characteristics of BO-selective cells and was supported by psychophysical experiments (Sugihara et al., 2007; Sakai et al., 2012). Based on this mechanism for determining the BO, we proposed a model of attention for the modulation of BO-selective cells (Wagatsuma et al., 2008, 2013a). The responses of these models showed good agreement with human perception for the modulation magnitude. However, the time-course of the responses by BO-selective model cells was not analyzed or discussed herein. In the present study, in order to clarify the roles of the feedforward signals based on surrounding suppression/facilitation and the cortical interactions between early and parietal visual areas for the time-course of BO-selective cells, we used the same cortical network employed in our previous models and then analyzed the dynamics of the current network model.

**Figure 1:**
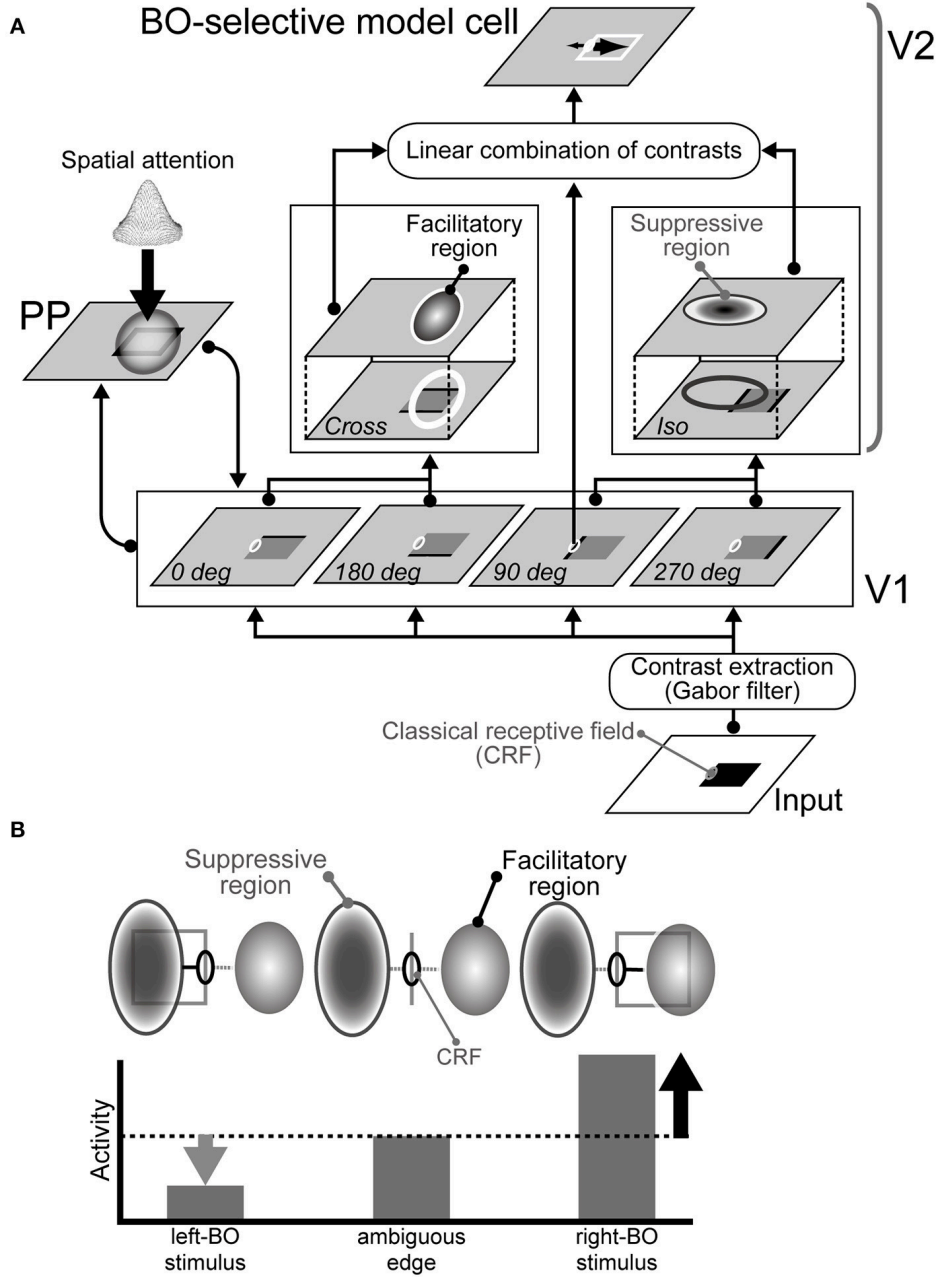
**Proposed network model. (A)** Architecture of the model comprising three modules: V1, V2, and PP (Wagatsuma et al., 2008). The activities of the BO-selective model cells in the V2 are based on surrounding luminance contrast extracted by the V1. Spatial attention represented in the PP enhances contrast gain in the V1. **(B)** Illustration of the mechanism for right BO-selective model cells (Sakai and Nishimura, 2006; Sakai et al., 2012). This cell has facilitatory and suppressive regions on the right and left of the CRF, respectively. When a bar is projected onto the CRF of the model cell, the cell responds to some degree, as shown at the center. If a figure (square) falls on the right side from the CRF, then the figure's contrast within the surrounding facilitatory region excites the activity of the cell (right). However, if a figure falls onto the suppressive region, the activity is inhibited (left). Therefore, the activity of the cell is higher activity when a figure is placed on the right of the CRF, thereby indicating right-BO selectivity.

Our current model consists of three modules, i.e., V1, V2, and PP modules, as illustrated in Figure 1A (also see Wagatsuma et al., 2008). In our model, weak modulatory feedback representing the responses of the PP module and mediating the spatial attention increase contrast gain in V1, which then modulates the responses of V2 that determine BO from the surrounding suppression/facilitation based on low-level features (Figure 1B). V1 and PP interact with each other to allow the application of attention in V1 and to modify attention in PP. Our model (Wagatsuma et al., 2008) and previous computational studies (Rolls and Deco, 2002; Deco and Lee, 2004) shared the dorsal pathway presented by the V1-PP network. Especially, Deco and Lee (2004) implied that the PP module had an important role for demonstrating the visual search capability and computing object location in the spatial domain.

Each module is comprised of 100 × 100 model neurons positioned retinotopically. In our previous study (Wagatsuma et al., 2008), we introduced dynamics into the model because V1 and PP are mutually connected to include weak feedback. Therefore, the activities of the model cells are represented by a partial differential equation with time (*t*) and space (*x* and *y*) variables. In the following equations, which focus on dynamic change, we omit the space variables (*x* and *y*) or represent them as if they are constants. When there were no external inputs, the activity of a neuron at time *t, A(t)*, is given as follows:

(1) $$\begin{array}{l} \tau \frac{\partial A\left(t\right)}{\partial t}=-A\left(t\right)+\mu F\left(A\left(t\right)\right), \end{array}$$

where −*A(t)* on the right side represents decay, and μ*F (A(t))* considers the excitatory, recurrent signal among the excitatory neurons. The non-linear function, *F(x)*, is given as follows:

(2) $$\begin{array}{l} F\left(x\right)=\frac{1}{T_{r}-\tau \;\log\left(1-\left(\frac{1}{\tau x}\right)\right)}, \end{array}$$

where τ is a membrane time constant (10.0 ms) and *T*_*r*_ is the absolute refractory time (0.5 ms). The Equation 2 is the response function for transforming current into discharge rate for a spiking neuron with deterministic input (Rolls and Deco, 2002). These two terms of Equation (1) on the right-hand side are necessary for discussing the time-course of the proposed model. The dynamics of this equation and the appropriate values for the constants have been studied widely (e.g., Gerstner, 2000).

The present model mechanisms and parameters were common to the previous spatial attention work (Wagatsuma et al., 2008), except for the numerical method used to solve the differential equations. In the previous work, no other than the magnitudes of the activities of BO-selective model cells were analyzed. In order to discuss the temporal characteristics of the responses by BO-selective cell, we now use a more accurate numerical method. Here, we integrated the differential equations using a standard fourth-order Runge-Kutta algorithm with a time step of 0.1 ms. The code for our simulations was written in MATLAB.

### V1 module

The V1 model cells represent the local, oriented contrast from input stimuli via convolution of the image with a set of Gabor filters with four orientations. The response of the model cells is determined by convolution with the visual input, the previous response of the cell, and weak feedback inputs from the PP. The extracted local contrasts from input stimuli have an intensity value ranging between zero and two, which are modulated by feedback from the PP module (see the Supplementary Material). In our model, the connection weights of feedback signals are markedly weaker than that of feedforward inputs (Deco and Lee, 2004). Previous physiological work has also reported that the efficacy of the feedback stimulation for inducing a postsynaptic activity is smaller than that of the feedforward connection (Salin and Bullier, 1995).

The activity of a model V1 cell, $A_{\theta \omega xy}^{V1}\left(t\right)$, is given as follows:

(3) $$\begin{array}{l} \tau \frac{\partial A_{\theta \omega xy}^{V1}\left(t\right)}{\partial t}\;=\;-A_{\theta \omega xy}^{V1}\left(t\right)\;+\;\mu F\left(A_{\theta \omega xy}^{V1}\left(t\right)\right)\;+\;I_{\theta \omega xy}^{V1,exc}\left(t\right)\\ +\;I_{noise}^{V1}\left(t\right), \end{array}$$

where *x* and *y* show spatial locations, θ and ω are the preferred orientation and spatial frequency, respectively, $I_{noise}^{V1}$ represents uniformly distributed random noise between −0.25 and 0.25, and μ represents the scaling constant (μ = 0.95 was used). The activities of the model V1 cells are modulated by feedback from the PP module in an exponential manner, as proposed previously (Lee et al., 1999; Peters et al., 2005), which is shown by $I_{\theta \omega xy}^{V1,exc}\left(t\right)$. This exponential modulation acts on divisive normalization where the luminance contrast at a location is divided by the spatial pool of neighborhood contrasts, as described by Equation (S2) in the Supplementary Material. This normalization plays a role of an inhibitory mechanism, which is crucial for the stability during recurrent computation. According to this mechanism, top-down attention increases the low-level feature so the contrast gain at the attended location is enhanced in the model. The feedback from the PP module is 0.6 of the feedforward connection in weight (see the Supplementary Material and Deco and Lee, 2004). The activity of this model cell represents the response of a V1 model cell to the stimulus projected onto its CRF. A detailed mathematical description is shown in the Supplementary Material (also see Wagatsuma et al., 2008).

### V2 module

The V2 module comprises BO-selective model cells, which determine the BO based solely on the contrast signals that surround their CRF, which are extracted by V1, as illustrated in Figure 1B. Each BO-selective model cell has facilitatory and suppressive regions, the location, shape, and size of which determine the selectivity of the cell. The activity of a model V2 cell, $A_{xyN}^{V2,BO}\left(t\right)$, is given as follows:

(4) $$\begin{array}{l} \tau \frac{\partial A_{xyN}^{V2,BO}\left(t\right)}{\partial t}\;=\;-A_{xyN}^{V2,BO}\left(t\right)\;+\;\mu F\left(A_{xyN}^{V2,BO}\left(t\right)\right)\\ -\;\gamma F\left(A^{V2,inh}\left(t\right)\right)\;+\;I_{xyN}^{V2-V1,BO}\left(t\right)\;+\;I_{noise}^{V2}\left(t\right), \end{array}$$

where *N* represents the type of BO-selective model cell, which is defined by the surrounding facilitatory/suppressive regions. An index *BO* represents the BO selectivity where, for the sake of simplicity, we restricted our analysis to either the left or the right in order to consider only vertical borders in the simulations. If the activities of left BO-selective model cells are higher than that of right BO-selective cells, then the DOF is determined as left. Moreover, $I_{noise}^{V2}$ indicates uniformly distributed random noise ($-0.25\leq I_{noise}^{V2}\leq 0.25$), and γ represents the scaling constant (γ = 0.8 was used). Again, this V2 module receives only feedforward inputs which were mediated by surrounding suppression/facilitation. In the current work, we did not implement the direct feedback signals from PP to V2 modules.

The activity of $I_{xyN}^{V2-V1,BO}$ is determined by retinotopical, feedforward signals from V1, including the surrounding low-level features such as the contrast (Sakai and Nishimura, 2006):

(5) $$\begin{array}{l} I_{xyN}^{V2-V1,BO}\left(t\right)=O_{xy}^{1}\left(t\right)\left(O_{xy}^{1}\left(t\right)+O_{xyN}^{2,BO}\left(t\right)\right), \end{array}$$

where

(6) $$\begin{array}{l} O_{xyN}^{2,BO}=CF_{xyN}^{BO}-CS_{xyN}^{BO}. \end{array}$$

A detailed mathematical description is given in the Supplementary Material. $O_{xy}^{1}$ is the feedforward input from the V1 module, which corresponds to the CRF responses. $O_{xyN}^{2,BO}$ is the contrast surrounding the CRF. $I_{xyN}^{V2-V1,BO}$ is based on the summation of CRF response, *O*^1^, and the surrounding response, *O*^2^, which represents the surrounding suppression/facilitation apparent in the early visual areas (Jones et al., 2001, 2002). Multiplying by *O*^1^ acts as a switch so a response is not observed when there is no stimulus on the CRF. $CF_{xyN}^{BO}$ and $CS_{xyN}^{BO}$ are facilitatory and suppressive surrounding contrast signals, respectively, which are determined by the spatial convolution of the V1 responses and the corresponding surround regions with a Gaussian shape, as illustrated in Figure 1B. A wide range of BO selectivity has been reported in physiological experiments (Zhou et al., 2000), but we selected 10 types of surrounding regions from a pool of randomly generated Gaussians, which induced the robust and consistent determination of BO for the square (Sakai and Nishimura, 2006; Sakai et al., 2012). Because it is intuitive to include this surrounding suppression/facilitation in the V1 module, we also included this process in the V2 module for simplifying computation. It has also been reported that V2 neurons exhibit similar surrounding suppression/facilitation (Ito and Komatsu, 2004).

In Equation (4), *A*^*V*2, *inh*^ represents the activity of an inhibitory neuron. We implemented a single inhibitory unit for each of the V2 and PP modules to limit the activities of the module within a certain range. The activity of the inhibitory model cell for V2 is given as follows:

(7) $$\begin{array}{l} \tau \frac{\partial A^{V2,inh}\left(t\right)}{\partial t}\;=\;-A^{V2,inh}\left(t\right)\;+\;\lambda F\left(A^{V2,inh}\left(t\right)\right)\\ +\;\kappa \underset{x,y,N,BO}{\sum }F\left(A_{xyN}^{V2,BO}\left(t\right)\right), \end{array}$$

where κ and λ are scaling constants (κ = 0.05 and λ = 0.1 were used). The inhibitory neuron receives inputs from excitatory neurons and inhibits all of them.

### PP module

The PP module represents the location of visual objects and the allocation of attention. This module is designed to represent spatial information and domain based on the luminance contrast. In our model, synaptic weights of feedback connection are markedly smaller than that of feedforward (Supplementary Material). However, this weak modulatory feedback from the PP module facilitates the contrast processes in the V1 module for the presented object and in the attended location. The PP module indicates where bottom-up attention from the responses of the V1 module and the top-down spatial attention should be directed. The activity of a PP model cell, $A_{xy}^{PP}$, is given as follows:

(8) $$\begin{array}{l} \tau \frac{\partial A_{xy}^{PP}\left(t\right)}{\partial t}\;=\;-A_{xy}^{PP}\left(t\right)\;+\;\mu F\left(A_{xy}^{PP}\left(t\right)\right)\;-\;\gamma F\left(A^{PP,inh}\left(t\right)\right)\\ +\;I_{xy}^{PP-V1}\left(t\right)\;+\;I_{xy}^{PP,A}\left(t\right)\;+\;I_{noise}^{PP}\left(t\right), \end{array}$$

where $I_{xy}^{PP,A}$ represents the bias of spatial attention, which is given by a Gaussian with a simplified shape (Müller et al., 2005; also see **Figures 6**, **7**), and $I_{xy}^{PP-V1}$ represents afferent signals from the V1 to PP. These two inputs represent the object location and they determine the strength of attention. *A*^*PP, inh*^ denotes an input from an inhibitory PP neuron, whose activity is given in a manner similar to that in Equation (7). Finally, $I_{noise}^{PP}$ represents uniformly distributed random noise ($-0.25\leq I_{noise}^{PP}\leq 0.25$).

## Results

We investigated the roles of feedforward signals and cortical interactions for the temporal characteristics of physiology and psychophysics in BO determination. Specifically, we examined the computational model that comprised of V1, V2, and PP modules (Figure 1; Wagatsuma et al., 2008) in order to discuss the dependence of time course on the ambiguity of DOF in BO-selective cells and human response time in the corresponding psychophysics. Simulations of our proposed model were performed with a variety of visual inputs, which corresponded to the physiological and psychophysical stimuli (Posner, 1980; Wagatsuma et al., 2008; O'Herron and von der Heydt, 2009). The stimuli corresponding to physiological experiments (O'Herron and von der Heydt, 2009) were mainly used to examine the influences of the feedforward inputs for the time-course of BO-selective cell responses whereas we used the psychophysical stimuli (Posner, 1980; Wagatsuma et al., 2008) to study the roles of the V1-PP interactions for the response time of figure perception. To analyze the results of these simulations, we computed the BO signals ν (O'Herron and von der Heydt, 2009), which were defined by the difference in the population activities of the left and right BO-selective cells:

(9) $$\begin{array}{l} \nu \left(t\right)=\underset{N}{\sum }A_{xyN}^{V2,Left}\left(t\right)\;-\;\underset{N}{\sum }A_{xyN}^{V2,Right}\left(t\right). \end{array}$$

Positive ν indicates the dominance of the left BO-selective population, while negative ν-values indicate dominance of the right population.

### Time-course of the BO signal in the proposed model

**Figure 2:**
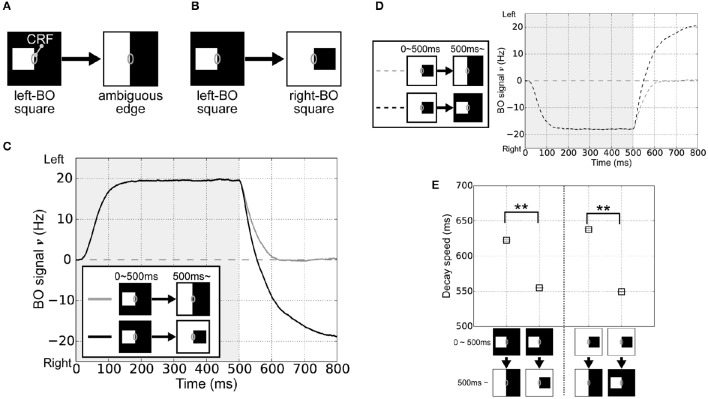
**Visual stimuli in the corresponding physiological experiment (O'Herron and von der Heydt, 2009) and V2 module responses. (A)** Schematic representations of the stimuli used in the simulations; where the gray ovals indicate the CRF. The right edge of a white square was first presented in the CRF of BO-selective model cells (time < 500 ms) and then switched to an ambiguous edge (time > 500 ms). **(B)** The other stimulus used in the simulations. On the CRF of the BO-selective model cells, the first figure (right edge of a white square: time < 500 ms) was replaced by a second (left edge of a black square; time > 500 ms). **(C)** Time-course of the average BO signal ν (based on 20 simulation trials). Gray and black lines indicate the time-course of BO signals ν for stimulus sets **(A,B)**, respectively. The BO signal ν for the stimulus set **(B)** without ambiguity was modulated more rapidly than that was for set **(A)**, which included the ambiguous edge. **(D)** Time-course of the BO signal ν when the right-BO square was presented as the first figure. **(E)** Neural decay speed of the BO signal ν. Asterisks indicate significant differences between the stimulus sets (*t*-test: ^**^p < 0.01; ^*^p < 0.05).

In order to investigate the roles of the integration of feedforward signals via surrounding suppression/facilitation for the time-course of the responses of BO-selective cells in V2 module, we performed simulations of the model with visual stimuli that corresponded to physiological experiments performed by O'Herron and von der Heydt (2009). In their experiments, a single square was presented with its right edge aligned along the CRF of the BO-selective cell. The square was then enlarged so a clear DOF (left) was changed into an ambiguous DOF at the midline of the screen's center (Figure 2A). Here, the firing rates of BO-selective cells decreased slowly after switching to an ambiguous edge compared with switching to a clear DOF, as observed when flipping the square with respect to the CRF (Figure 2B).

To test the time-course of the BO signal ν in our model, the right edge of a white square was first given in the CRF of the BO-selective model cells (left-BO square, time 0–500 ms), followed by its replacement with an ambiguous edge (Figure 2A) or the left edge of a black square (right-BO square, Figure 2B) at 500 ms. Figure 2C summarizes the mean BO signal ν for these visual stimuli based on 20 simulation trials. During the first figure presentation (0–500 ms), we obtained positive BO signals ν after a steep rise, which indicates that the left BO-selective populations were markedly more excited than those on the right were. When the first square was switched to the right-BO square (>500 ms), the BO signals ν decayed more rapidly (black line in Figure 2C) compared with that after replacement by the ambiguous edge (gray line in Figure 2C). When we reversed the sides of the squares so that the right-BO square was presented first (Figure 2D), we observed negative BO signals ν but the identical characteristics of the time course; slow decay after replacement with the ambiguous edge (gray dashed line in Figure 2D) and rapid modulation with the opposite clear DOF (black dashed line in Figure 2D). The time course of the BO-selective model cells was modulated by the DOF ambiguity of the visual inputs similar to, in a qualitatively manner, physiological observations (O'Herron and von der Heydt, 2009).

To quantify these simulation results, we computed the decay speed of the BO signals with respect to these stimulus sets. We used the biological time when the BO signals ν arrived 0 Hz as the index of neural decay speed. This index indicates the biological time when there was a similar level of population activities between left and right BO-selective cells. The decay speed indices are shown in Figure 2E. We observed the significant differences in the decay speed of the BO signals depending on the stimulus sets (*t*-test, *p* < 0.01). This ambiguity dependence is caused by the integration delay of V1 and V2 cells and the local inhibition therein given the difference in input stimulus. For further analyses of these simulation results, we fitted functions to BO signals ν of each simulation trial and calculated their slopes after the replacement (Figure S1A). The slopes corresponded to the magnitude of the signal change per second (O'Herron and von der Heydt, 2009). When the first square was flipped, these slopes were significantly higher compared to that after the replacement by the ambiguous edge. To analyze the BO signal decay speed based on both slope and time constant, we computed the absolute values of the derivative for exponential curves (Figures S1B,C), which indicated the speed of the BO signals ν in our model. During time 500 and 800 ms, the derivatives under after the replacement with the opposite clear DOF were consistently higher than that with the ambiguous edge. These results indicated the ambiguity dependence of the time-course in our model. Since the responses of the BO-selective model cells were determined solely by the luminance contrast extracted by the V1 module (Figure 1), our model suggests the contribution of early-level stimulus features to the ambiguity dependence of the time course. The detailed examination is given in the next section.

In our model, although the decay speeds were modulated, the integration delay and the local inhibition do not fully explain the ambiguity dependence of time course. The physiological BO signals indicated the persistent activation several times longer than our simulations after the replacement with the ambiguous edge. Additional mechanisms appeared to be necessary to fully reproduce the distinct time-course depending on the DOF. A possible candidate to fill the gap between the two is the slow feedback projections to BO-selective cells that could be mediated by NMDA and is absent from the model. This possibility will be discussed further in the Section Discussion. In the following sections, we look into the dynamics of our model to discuss the plausible mechanisms that explain the temporal characteristics of physiology and psychophysics in BO determination.

### Time-course of the responses by V1 and PP modules when detecting a new object

The simulations of the model exhibited the time-courses depending on the BO ambiguity. However, the transition time of the physiological BO signals was several times longer than that of our model signals. Here, we examined the responses of our model and discuss the neural mechanisms underlying the ambiguity dependence. In the model, surrounding luminance contrasts extracted in the V1 underlie the activities of BO-selective model cells in the V2 module. The model also has cortical interactions between V1 and PP modules, which has been reported to be important for the determination of BO (Wagatsuma et al., 2008). The feedback from PP representing the location of the visual stimulus facilitates the early-level features and modulates the responses of BO-selective model cells. These mutual interactions between V1 and PP modules might have the influences on the time-course of BO-selective model cells. To understand the influence of these cortical interactions to the time-course of BO signals ν, we examined the time-course of the responses in the V1 and PP modules when a second stimulus was presented (Figure 2B).

**Figure 3:**
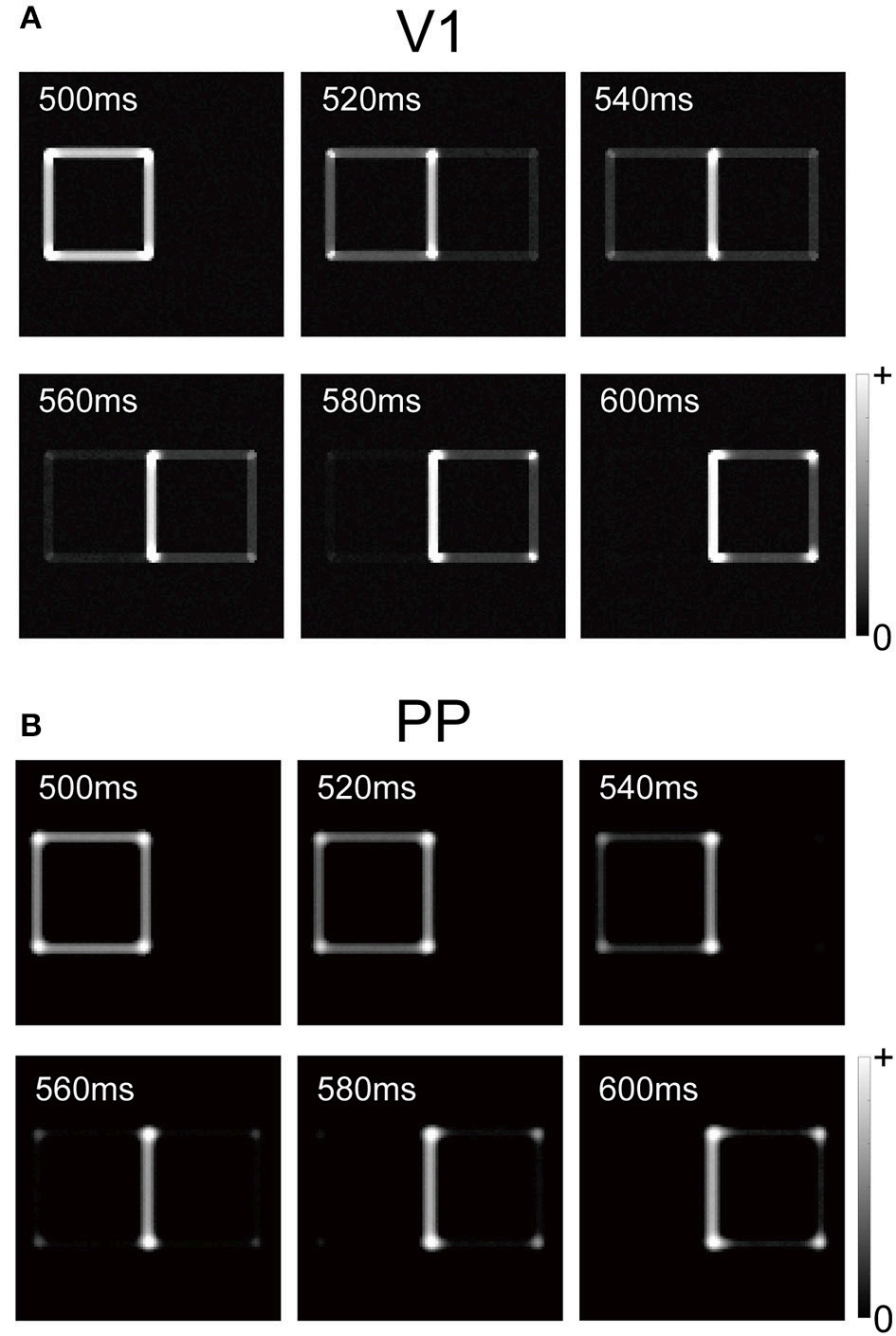
**Responses of V1 and PP modules with respect to biological time (500–600 ms) for the stimulus corresponding to Figure 2B**. We changed a left-BO white square to a right-BO black square at 500 ms (biological time). **(A)** Responses of the V1 module. All orientation factors in the V1 module were merged for visualization. Between 540 and 560 ms, the V1 module responded to the luminance contrasts for both the left- and right-BO squares. **(B)** Responses of the PP module. In contrast to V1 responses, regardless of right-BO black square presentation, the PP module did not exhibit sufficient response to the new figure until 580 ms in the simulation.

Figure 3 summarizes the time-course of the responses by the V1 and PP modules to the visual input after replacing the left-BO square with a right-BO square (time 500–600 ms in Figure 2B). To focus on the responses to the second square, we showed the time-course after 500 ms in the figure. From 540 to 560 ms, the V1 module responded to the luminance contrast of the second figure but it also preserved that of the first (Figure 3A). Notably, during this period, the BO signals ν reached a level of 0 Hz (Figure 2C). This result indicates that the neural dynamics (integration delay) and the latency of feedforward inputs result in the time-courses based on input stimuli, suggesting the contribution of early-level stimulus features to the ambiguity dependence.

In contrast to the V1 module, the presentation of the second figure was not sufficient to reverse the activation of the PP evoked by the first figure. Compared to the PP responses at 500 ms, whereas the cells in the PP at 580 ms were activated around the corners of the second figure, only a small part of their edges were detected (Figure 3B). Note that, for the V1 module, the all edges of the second square had been presented at 580 ms, although their responses at this time were weaker than that at 500 ms. In our network model, the activation of the PP module after the newly presented figure followed that of V1. In addition, the PP module preserved the response to the first stimulus after 540 ms, which may influence the responses of the V1 module via the feedback. A possible hypothesis is that a lack of inhibition of return (Itti and Koch, 2001) prevented our model from fully detecting the new figure and induced the gap between the stimulus presentation and these modules responses, as described in the Section Discussion. These results suggest that the ambiguity of the figure's direction, as well as the V1-PP network, played important roles for the time-course of BO-selective cells in the V2.

### Duration of figure presentation and BO signal persistence

O'Herron and von der Heydt (2009) performed physiological measurements of the decay of BO signals ν during figure presentation for various durations. Interestingly, the persistence of the signal in the ambiguous edge phase was independent of the figure presentation duration. The feedforward signals in the visual system might be influenced by the figure presentation duration. To examine the influence of the figure presentation duration on the decay of the BO signal ν in our current model, we performed model simulations with five figure presentation durations, i.e., 500, 250, 125, 63, and 50 ms.

**Figure 4:**
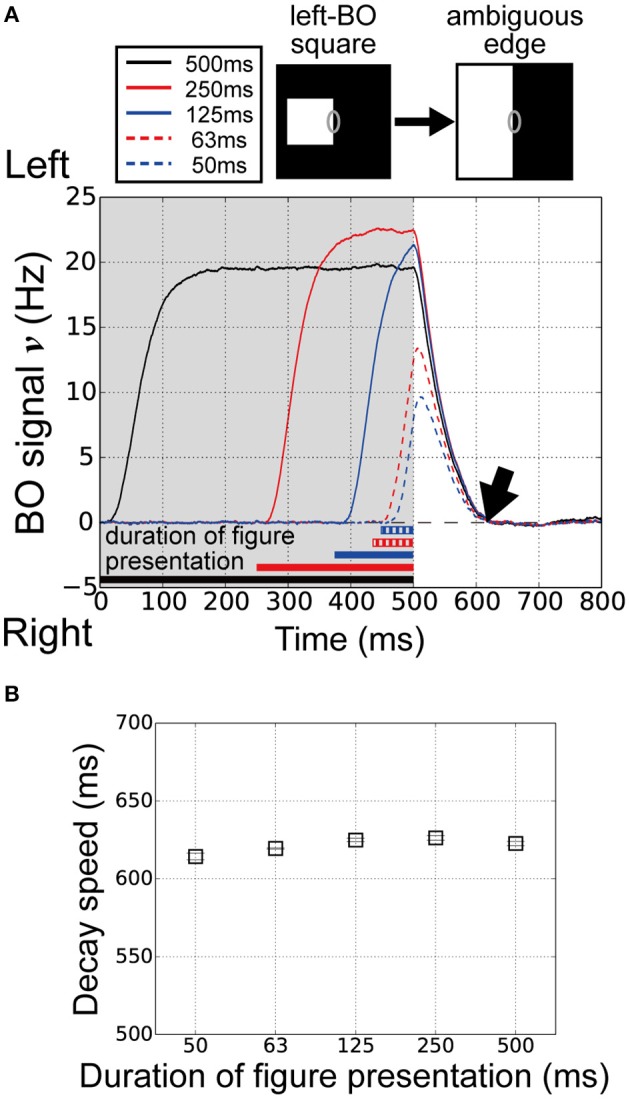
**Duration of figure presentation to examine BO signal ν persistence. (A)** Time-courses of the BO signals ν for various durations of figure presentation (black, 500 ms; solid red line, 250 ms; solid blue line, 125 ms; dashed red line, 63 ms; dashed blue line, 50 ms). The convergence times of the BO signals ν were similar for all durations (arrow). **(B)** Average decay speeds of the BO signal ν for five figure presentation durations (20 trials). Error bars represent the standard error. Despite the marked differences in figure presentation duration, the decay speed was similar at each duration.

The time-courses of the BO signal ν over five different durations of figure presentation are shown in Figure 4A. Durations of 125 ms (blue solid line) and 250 ms (red solid line) produced signals with a higher magnitude than that of 500 ms (black). We observed higher responses on V1 module for the duration of 125 and 250 ms than that for 500 ms (data not shown). In contrast, at durations of 50 ms (blue dashed line) and 63 ms (red dashed line), the signals showed markedly lower signal magnitude than the other three durations did. However, the persistence of the signal in the ambiguous edge phase was similar at all five durations (Figure 4A, arrow). To examine the performance of our model in more detail, we computed the average decay speed for the BO signal ν in the ambiguous edge phase (time > 500 ms) for each of the five figure presentation durations (Figure 2E). Figure 4B shows the decay speeds of the BO signals ν. Regardless of the marked differences in the magnitude of the BO signal ν, the decay speed was similar at all five durations (duration 500:622.7 ms; duration 250:626.2 ms; duration 125:624.9 ms; duration 63:619.5 ms; duration 50:614.3 ms). Thus, the results of our current model imply that the decay speed is duration-independent. In our proposed model, the responses of the BO-selective model cells in the V2 were determined by feedforward inputs, as well as by the V2 inhibitory unit. These results suggest that the V1–V2 feedforward network and the local inhibition underlie the time-course of the BO signal ν.

### Responses of BO-selective model cells across repeated figure presentations

**Figure 5:**
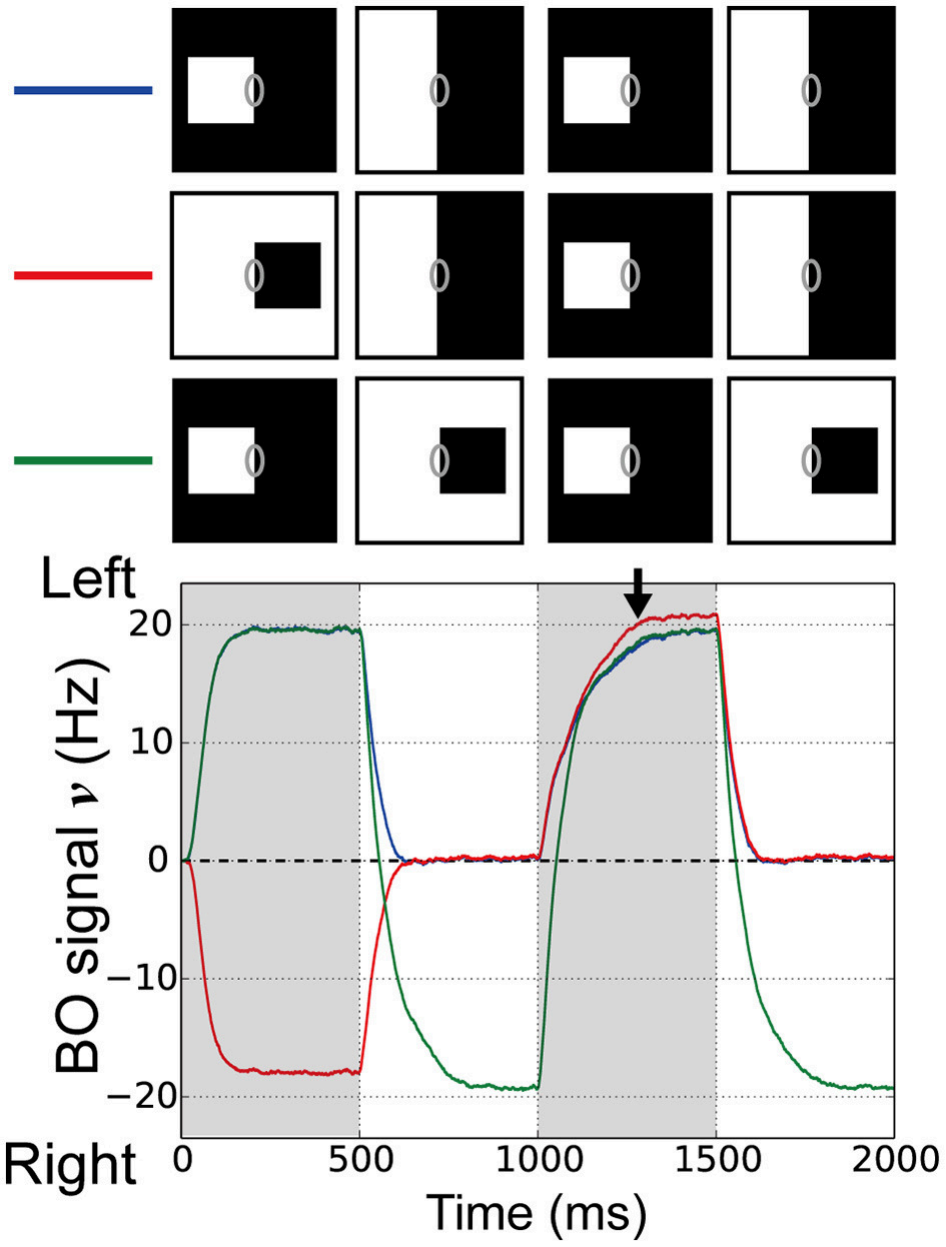
**Responses of BO-selective model cells in the V2 module with repeated figure presentation**. Simulations of our model were performed using three stimulus conditions. In two conditions, the figures were presented twice with either the same DOF (blue line) or opposite DOF (red line), and each presentation was followed by 500 ms of ambiguous edge display. In the third condition, the figure was switched without presenting the ambiguous edge (green line). Stimuli presentation sequences are shown at the top. In all three conditions, the left-BO white square was presented as the third stimulus (1000–1500 ms). It should be noted that all three BO signals converged during this interval (arrow). Each new figure presentation resets the BO signal in our model, which agreed with physiological observations.

Next, we examined the influences of repeated figure presentation using our proposed model. The responses of BO-selective cells *in vivo* do not accumulate over repeated figure presentations, i.e., each newly presented figure resets the signal (O'Herron and von der Heydt (2009) and see their Figure 4), which seemed to imply the effects of the feedforward signals for the time-course of BO signals. To examine the responses of BO-selective model cells to the repeated figure presentation, we performed simulations where the figures were presented twice, followed by ambiguous edge presentation at 500 ms (Figure 5, blue and red lines). In these simulations, the left-BO square was presented as the third stimulus presentation (time 1000–1500 ms), which appeared either on the same side as the first figure (Figure 5, blue line) or on the opposite (Figure 5, red line). During the first figure presentation (time 0–500 ms), there was a clear difference in the magnitude of the BO signal ν between the two conditions. However, regardless of this clear difference at the onset of the ambiguous edge (time 500 ms), these signals ν converged at almost the same time (time 500–1000 ms) and exhibited a similar time-course during the third stimulus presentation (time 1000–1500 ms). In the third condition, the left-BO square was presented as the first stimulus (time 0–500 ms) and the third stimulus (time 1000–1500 ms) was replaced by the opposite-BO square instead of the ambiguous edge (Figure 5, green line). In this case, the BO signal ν was strongly negative at the beginning of the third stimulus presentation (time 1000 ms), but during the third stimulus (time 1000–1500 ms), the signal reached a similar amplitude to that in the other two conditions (black arrow in Figure 5). These results imply that the BO signal ν generated by our model was reset by each newly presented stimulus. Our model suggests that each new stimulus presentation underlying the feedforward signals from V1 to V2 may play important roles in the time-course of the BO signal ν.

### Spatial attention in early vision to modulate the time-course of the BO signal

Our proposed model implied the roles of the feedforward signals from V1 to V2 cells and the V1-PP network for the ambiguity dependence of the time-course on BO signals. Several studies have indicated that selective attention, as well as visual stimuli, can modulate the responses of BO-selective cells (Qiu et al., 2007; Martin and von der Heydt, 2015) and object perception (Hasson et al., 2001; Vecera et al., 2004; Pitts et al., 2007). Furthermore, our previous studies have shown that selective enhancement of low-level feature contrast by attention underlies figure-ground flipping (Wagatsuma et al., 2008, 2013a). However, the effects of such selective enhancement in low-level vision on the time-course of the BO signal were not investigated. The attentional effect for the time-course of the BO signals in our model might give an insight into the modulation of visual perception. Thus, we explored the roles and effects of spatial attention in an early vision to modulate the time-course of the BO signal ν and the response time for visual perception. In addition to the bottom-up visual input, top-down spatial attention was applied to the PP module to enhance the representation of the stimulus location and the luminance contrast in V1 (see Materials and Methods, Supplementary Material and Figure 1A).

**Figure 6:**
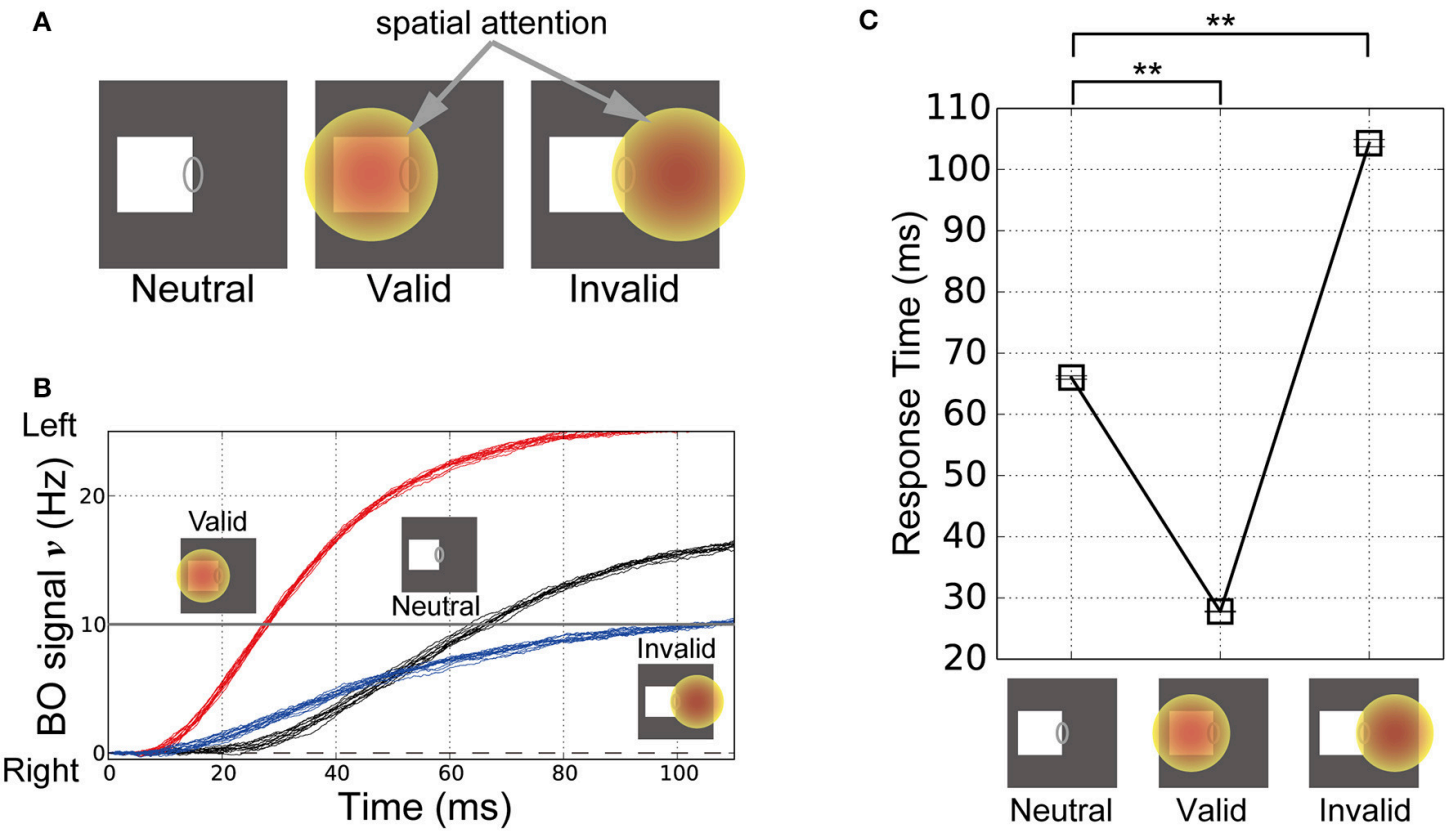
**Stimuli in the corresponding classical psychophysical experiments (Posner, 1980) and time-course of the BO signal υ. (A)** Stimuli and locations of spatial attention in the simulations of Posner's experiments. In these simulations, the right edge of a white square was presented in the CRF of BO-selective model cells. In the “Neutral” condition, spatial attention was not given to the model (left panel). In the “Valid” condition, there was spatial attention at the center of the presented square (center panel). In the “Invalid” condition, spatial attention was applied outside the square in the opposite direction relative to the CRF (right panel). **(B)** Time-courses of the BO signals ν for the stimuli corresponding to **(A)** based on 20 simulation trials. Black, red, and blue lines indicate the time-courses of the BO signals ν for the Neutral, Valid, and Invalid conditions, respectively. The three icons at the top represent the three simulation conditions. **(C)** Mean response time of our model when the white square was presented (20 trials). Error bars represent the standard error, which are small. Response time was defined as the biological time when the BO signal exceeded a level of 10 Hz. Asterisks indicate significant differences between locations of spatial attention (*t*-test: ^**^p < 0.01; ^*^p < 0.05). The location of spatial attention significantly modulated the response time for visual perception.

To explore the influence of spatial attention in modulating the time-course of the BO signal ν, we performed simulations of our model based on visual stimuli and attention in corresponding classical psychophysical experiments (Posner's experiments; Posner, 1980). In these simulations, the left-BO square was given in the CRF of the BO-selective model cells. Figure 6A illustrates the three simulation conditions in Posner's experiments, where spatial attention was applied nowhere (Neutral, left panel in Figure 6A), at the center of the square (Valid, center in Figure 6A), and outside the square in the opposite direction from the square relative to CRF (Invalid, right in Figure 6A). Posner has indicated that, under the Valid condition, the response time of human participants for the target detection was significantly decreased compared to the Neutral condition. By contrast, under the Invalid condition, the response time was increased.

The time-courses of the BO signals ν in these three conditions are shown in Figure 6B. In all cases, the activities of the left BO-selective model population were dominant over those of the right BO population; therefore, our model robustly and consistently determined the presented square as the figure irrespective of the spatial attention location. Intriguingly, the time-course of the BO signal ν for the Valid condition (Figure 6B, red lines) increased more rapidly than that for the Neutral condition (Figure 6B, black lines). In contrast, there was a more moderate increase in the time-course of the signal under the Invalid condition (Figure 6B, blue lines). These simulations of our model suggest that spatial attention to the location of the presented stimulus facilitates the target detection, whereas attention to the outside of the stimulus suppresses it. These results are in qualitative agreement with attentional effects shown by psychophysical experiments (Posner, 1980).

In order to quantify the responses of the model to stimuli corresponding to Posner's experiments, we computed the response time required for perceiving the square. When the BO signal ν exceeded a level of 10 Hz, the biological time was treated as the response time of the model. Figure 6C summarizes the mean response time in the Neutral, Valid, and Invalid conditions based on 20 simulation trials. There were significant differences in the response times for the Neutral and Valid conditions (*t*-test, *p* < 0.01). Furthermore, the Invalid condition had a significantly longer response time compared with the Neutral condition (*t*-test, *p* < 0.01). In our model, spatial attention modulated not only the magnitude of the responses by BO-selective model cells, but also the activation speed of BO signal ν, which suggests that the location of spatial attention underlies the marked modulation of the response time for detecting the presented target. Intriguingly, the attentional facilitation and suppression related to the response time according to our model simulations agreed well with a previous psychophysical study of attention-based modulation of visual perception (Posner, 1980). Our model qualitatively and quantitatively reproduced the characteristics of human perception, which support strongly our hypothesis on the attentional mechanism.

**Figure 7:**
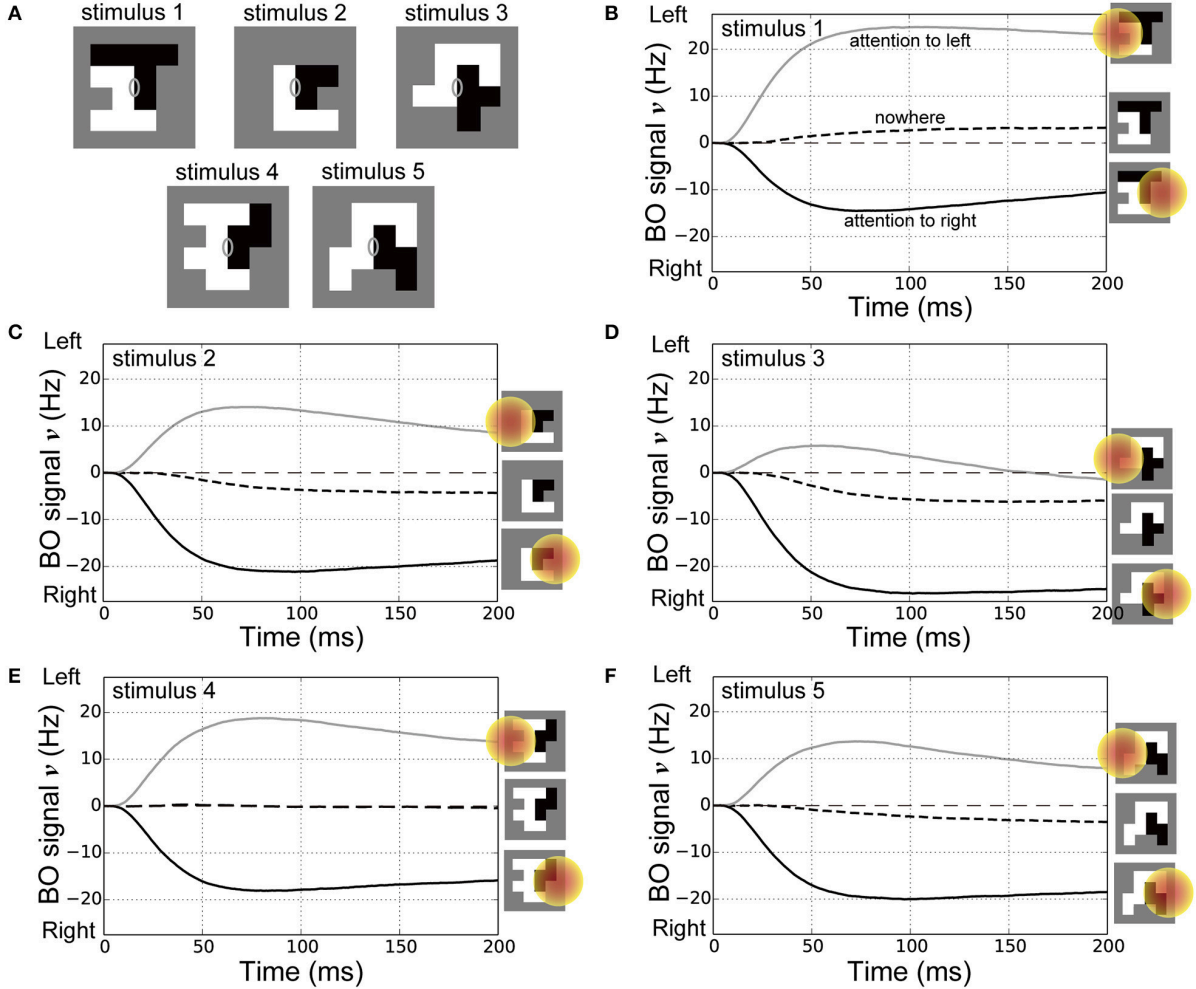
**Stimuli for corresponding psychophysical experiments (Wagatsuma et al., 2008) and time-courses of the BO signal υ. (A)** Five types of random-block stimuli with an ambiguous DOF at the center of the stimulus. Locations of the CRF and spatial attention in these simulations were identical to those in our previous experiments. **(B–F)** Time-courses of the BO signals ν for stimulus 1, 2, 3, 4, and 5 (20 simulation trials), respectively. Icons at the right show the location of spatial attention. Black dashed, gray solid, and black solid lines represent the BO signals ν without attention, with spatial attention to the left side, and with attention to the right, respectively. For all five stimuli, compared with the nowhere condition, spatial attention attracted the direction of BO toward the attended location and reduced the onset of the time-course for the BO signal ν.

Attention can even alter the perception of the DOF as demonstrated by ambiguous figures (Hasson et al., 2001; Pitts et al., 2007). In addition, the response times for reporting the presented figure were improved depending on the location of spatial attention (Vecera et al., 2004). These psychophysical works implied that attention modulated not only the responses of BO-selective cells but also the time-course of BO signals with respect to the ambiguous figures. To examine the effects of spatial attention in early vision for modulating the time-course of the BO signal ν with bi-stable figure perception, we performed simulations of the model with the ambiguous figures used in our previous psychophysical study (Figure 7A; Wagatsuma et al., 2008). These stimuli used in Figure 7A consisted of two adjacent random blocks (Sakai and Nishimura, 2006; Sakai et al., 2012) so that their BO around their center appeared to be ambiguous (gray ellipses on each panel of Figure 7A). In our psychophysical experiment (Wagatsuma et al., 2008), a single random-block stimulus with ambiguous BO was presented with its center aligned to the screen center. We measured the apparent direction of BO at the screen center through a two alternative forced-choice paradigm in which participants were asked to indicate which side appears in front of the other. While the DOF of these stimuli were ambiguous at the center of the stimulus, the apparent direction of the BO was shifted toward the direction of attention for all stimuli presented. In this psychophysical experiment, the DOF of these random-block stimuli from the center of the stimulus were bi-stable depending on the location of spatial attention. We also tested the reproducibility of the behavioral data in addition to the physiological data for the first time.

In the current simulations, spatial attention was applied nowhere, to the left or right side of the presented stimuli (also see the icons on the right-hand side of Figures 7B–F). The time-courses of the BO signals ν for the ambiguous figures are shown in Figures 7B–F. Under the nowhere condition (black dashed lines in Figures 7B–F), the time-courses of the BO signals ν for all stimuli were observed near a level of 0 Hz, which meant that there was a similar level of response between left and right BO-selective model cells. Without spatial attention, our model did not determine the direction of BO at the center of these ambiguous figures. In contrast, spatial attention modulated the BO signal ν toward the side of the attended location (black and gray lines in Figures 7B–F). This model reproduced the bi-stable BO determination depending on the attentional location with respect to the center of these ambiguous figures. Interestingly, the BO signal onset time that left a level of 0 Hz was markedly later under the nowhere vs. the two attention conditions. Furthermore, as shown in our previous work (Wagatsuma et al., 2008), spatial attention in V1 seemed to facilitate activation of the BO-selective model cells in V2, which may have reduced the response time to figure perception. These attentional effects for the activation speed of BO signal appear to modulate the perceptual response time, as shown in the simulations with stimuli corresponding to Posner's experiments (Figure 6). These attentional effects on the time-course of the BO signals for ambiguous figures are the suggestion from our model at the present time. We further describe the activation speed of BO signals ν in terms of the perceptual response time in the Section Discussion. These simulation results provide a support for the proposed mechanism of attentional modulation. These results suggest that spatial attention in early vision and the selective enhancement of early-level stimulus features via the cortical interactions modulate the responses of BO-selective cells in intermediate-level areas as well as improve psychophysical performance for the perception of figures.

## Discussion

In this study, we proposed a computational model comprised of three modules, which represented the V1, V2, and PP cortical areas to explore the roles of the integration of feedforward signals via surrounding suppression/facilitation and the mutual interactions between early and parietal visual areas for the time-course of BO-selective cells in an intermediate-level area. To our knowledge, this is the first study for modeling the temporal characteristics of BO signals depending on the ambiguity of DOF. In the proposed model, mutual connections between the modules included both feedforward and weak feedback pathways, except those from PP to V2 (Wagatsuma et al., 2008). Furthermore, spatial attention increased responses of the V1 module to modulate the activities of BO-selective model cells in the V2 because their activities were determined by surrounding suppression/facilitation based on early-level features extracted in V1 (Sakai and Nishimura, 2006). We performed numerical simulations under the same conditions as those in a previous physiological experiment (O'Herron and von der Heydt, 2009). The decay speed of the BO signals in our proposed model was modulated by the DOF ambiguity of the visual inputs. This ambiguity dependence was induced by the integration delay of feedforward inputs to V2 and the local inhibition. In addition, regardless of the lack of feedback from PP to V2, the time-course of BO signals ν of our model agreed with the characteristics of behavioral data in terms of the attentional facilitation and suppression of response time (Posner, 1980). Attention in early vision might lead to modulation of the human perception through the hierarchy of the visual pathway. The results of our simulations with these psychophysical stimuli imply that the selective enhancement of early-level stimulus features due to the V1-PP interactions underlies the modulation for the time-course of responses by BO-selective cells. Our proposed model suggests that feedforward signals via surrounding suppression/facilitation and cortical interactions between early and parietal visual areas, at least in part, play important roles for the time-course of BO-selective cells in the intermediate-level area as well as the visual perception of figure-ground segregation.

The time-course of our BO-selective model cells exhibited a similar tendency to that of monkey cells in the intermediate-level areas in terms of the dependence on BO ambiguity. An inhibitory unit in each module (e.g., Equation 7) appears to play an important role in this time-course. In our model, the inhibitory unit received inputs from all excitatory neurons in the module and inhibited them, irrespective of the selectivity of the neurons. When the presented square was replaced by the ambiguous edge (Figure 2A), the activity of the inhibitory unit in V2 is decreased with decreasing population activity of both left and right BO-selective model cells, which leads to the slow delay of the responses of BO-selective cells. By contrast, when the square was flipped (Figure 2B), the activity of the inhibitory unit in this module was sustained with sustaining population activity of the BO-selective model cells responding to the newly presented square, which leads to the rapid decrease of the responses. The duration-independent decay speed of BO-signals (Figure 4) appears also to arise from the effects of the local inhibition.

The time-course of our BO-selective model cells was affected by the ambiguity of the BO for the presented stimulus. However, in the physiological experiments (O'Herron and von der Heydt, 2009), the BO signal decayed slowly over the next second when the square was replaced by the ambiguous edge, which was not fully reproduced by our proposed model. Plausible mechanisms to explain the persistent activation of the BO-selective physiological cells over the next second include direct feedback projection to the intermediate-level from higher visual areas. Computational studies (Craft et al., 2007; Mihalas et al., 2011) indicate that excitatory feedback projections to the V2 from V4 representing the objects or shapes could also reproduce the modulations in order to influence responses of BO-selective cells. In contrast, in our modeling study aimed at understanding the role of attention in early vision, we did not introduce a connection between the V2 and PP, thereby excluding the direct attentional modulation of BO-selective model cells. Our previous psychophysical studies support the crucial contributions of the low-level features extracted during early vision for DOF perception (Sugihara et al., 2007; Wagatsuma et al., 2008, 2013a; Sakai et al., 2012). However, further studies of feedback modulation are required to clarify the modulation for time-courses of the responses by BO-selective cells and of the DOF perception.

Interestingly, recent physiological investigations indicate that feedback projections mediating selective attention and representing grouping structure may modulate the responses of BO-selective cells directly in the V2 and V4 (Qiu et al., 2007; Martin and von der Heydt, 2015). However, little is known about whether direct feedback projections into the V2 may modulate the time-course of the BO signal. Thus, further analysis of attention modulation in intermediate-level areas is necessary to understand the mechanism responsible for the persistent activation of BO-selective cells.

Recent physiological experiments have clarified the detailed neural mechanisms involved in transmitting feedback from higher- to lower-level areas. In particular, Self et al. (2012) indicated that the feedback activity in V1 responsible for figure-ground modulation depends on N-methyl-D-aspartate (NMDA) synaptic receptors. Another physiological study (Herrero et al., 2013) showed that NMDA-based synapses were involved with the feedback projections needed for top-down attentional modulation. The onset of NMDA synaptic currents is fast (a few ms), but the decay of these currents is markedly slow (50–250 ms; Hestrin et al., 1990; Elizabeth and Ary, 1999; Wang, 1999. The slow decay of NMDA-based synapses may induce the persistence of neuronal activity over the next second as shown in BO-selective cells in V2. However, we did not consider the characteristics of synaptic type-dependent dynamics because our current model is rate-based and still rather abstract. The network model with spiking neurons such as integrate-and-fire neurons might be necessary for understanding the neural mechanism of the persistent activation by BO-selective physiological cells.

The PP module represents the object location in the spatial domain and the allocation of attention. In the previous model (Deco and Lee, 2004), the responses of this module showed good agreement with the human perception for a serial attentional search. However, in our model, the presentation of the second figure was not sufficient to reverse the PP activation evoked by the first figure (Figure 3). It is possible that the failure to detect a new square in the PP module may be an important factor related to the difference between our model and physiological studies in terms of the speed of BO signal modification. It is possible that our model could not fully respond to a new square with the opposite DOF due to a lack of inhibition of return (Itti and Koch, 2001) because this biased attention away from the cued location. In our model, detection of a new stimulus location could be impaired due to the sustained responses to the initial stimulus in the PP module. Therefore, the inhibition of return for spatial attention may play an important role in perceiving and detecting a new object when it is projected onto the retina. This suggests that the V1-PP network, where attentional modulation to the early visual areas occurs, is critical for the time-course of neurons in intermediate-level areas such as V2 and V4.

We carried out model simulations with various figure presentation durations (Figure 4A). In these simulations, figure presentation durations of 125 ms (blue solid line) and 250 ms (red solid line) produced higher magnitude of BO signals ν than that of 500 ms (black). These differences in signal magnitude appeared to arise from the noise in our model network. During the simulations of our model, random noise was always given to all model cells. For the figure presentation duration of 500 ms, we presented both noise and visual stimulus throughout simulations from the start of simulations to the end. In contrast, for figure presentation durations of 125 and 250 ms, while we presented noise from the start of simulations, we presented a visual stimulus shortly after the simulation/noise onset (250–375 ms). Therefore, at the onset of the visual stimulus, the responses of the model network for the durations of 125 and 250 ms were markedly different from that of 500 ms. Complex interactions between the current activities of the model cells and the strength of inputs might determine the magnitude of the BO signals in the model. These influences of noise were also observed in Figure 5 for the responses of our model to the right-BO black square (red line, time 0–500 ms and green line, 1000–1500 ms).

Several studies have reported that visual attention enhances perception in various aspects, such as spatial frequency and orientation discrimination, dominance in binocular rivalry, and contextual modulation (Ito et al., 1998; Lee et al., 1999; Posner and Gilbert, 1999; Carrasco et al., 2004; Mitchell et al., 2004; Tzvetanov et al., 2006; Ling et al., 2009). In particular, spatial attention controlled by a visual cue improves the response time for target detection (Posner, 1980). Interestingly, we found that the time-course of BO-selective model cells was also modulated by the location of spatial attention, in a similar manner to psychophysical observations (Figure 6). However, the response time of human participants to the presented stimulus occurred within 250–400 ms, which was markedly different from our simulation results (30–110 ms; see Figure 6C). Afferent transmission beginning in the low-level features should gradually establish perception as the signal progresses through the hierarchy of the visual pathway (Felleman and Van Essen, 1991). The responses of higher-level areas such as the parietal and inferotemporal cortices may underlie the final perception of the presented stimulus. However, we computed the response time based on the activities of the V2 module, which involved much earlier level vision than that of the parietal and inferotemporal cortices. In addition, for the sake of simplicity, we did not introduce a detailed synaptic delay between modules in our model. It is likely that the response time for visual perception strongly reflects the accumulated cortical delay. Thus, a more detailed model is necessary to understand the detailed mechanism related to the human response time.

We have shown the time-courses of the BO signals ν with the ambiguous figures used in our previous psychophysical studies (Figure 7; Wagatsuma et al., 2008): the direction of BO around their center was ambiguous. In the procedure of spatial attention experiment (Wagatsuma et al., 2008), participants had to report the perceived DOF through a two alternative forced-choice paradigm even if the apparent figure was not perceived. This disorder in DOF perception with respect to the ambiguous figure would be reflected in the delayed response time. Interestingly, our simulation results implied that the onset time of the BO signal υ under the nowhere condition (black dashed lines in Figures 7B–F) was markedly later than that under the two attention conditions (black and gray lines in Figures 7B–F), which suggested that it would take longer to fix the stable DOF with respect to the ambiguous figures if participants fixated on the center of the screen through an experimental trial. This attentional facilitation of the response time for ambiguous figures is a prediction from the simulations of our model. Intriguingly, another psychophysical experiment using ambiguous figures has implied that attention improved the response time for reporting the perceived figure (Vecera et al., 2004) although their experimental stimuli, procedures and tasks were different from our previous work. These suggested that the neural mechanism of attentional modulation for the time-course of the responses by BO-selective cells to ambiguous figures was, at least in part, captured by our model. The response time required to perceive the DOF by human participants will provide an important insight for clarifying the mechanism underlying the temporal characteristics of the responses by BO-selective cells.

The neural dynamics of the rate-based model and the appropriate values for constants have been studied widely (Gerstner, 2000; Layton et al., 2012, 2014). In this work, we used additive first order differential equations for the activity of neurons as shown in Equation (1), which were used by various computational models for studying temporal characteristics of cortical responses (Rolls and Deco, 2002; Deco and Lee, 2004; Kandel et al., 2012). However, this dynamics mathematically does not have upper and lower limits for the responses of model cells. In our simulations, the extracted local contrasts from the input image were normalized for having an intensity value ranging between zero and two (see Materials and Methods and Supplementary Material). Furthermore, each module included the inhibitory mechanisms (e.g., Equation 7 and Equation S2) as well as the excitatory model cells. The total balance of the normalization of the input intensity and of the integration of these excitatory and inhibitory signals seemed to prevent the activities of each model cell from being infinitely increased and decreased.

A variety of models have been proposed to account for the neural mechanisms of BO allocation and figure-ground segregation. Craft et al. (2007) and Mihalas et al. (2011) assumed the grouping cells representing the figure or shape based on the activities of BO-selective cells. Feedback projections from grouping cells in higher visual areas could underlie the modulation which influences the determination of BO in V2. In addition, the grouping cells mediated selective attention to BO-selective cells in their model. As discussed previously, direct feedback projections from higher cortical areas appear to account for the modulation mechanism of the neural activities and dynamics of BO-selective cells (Qiu et al., 2007; Martin and von der Heydt, 2015; Wagatsuma et al., 2016). The model presented by Zhaoping (2005) demonstrated that interactions within the V2 area implemented the selectivity of BO. A model proposed by Grossberg (2016) implied that local interactions between the V2 neurons play an important role in figure-ground segregation of both 2D surfaces and 3D objects. In contrast, in our model, interactions within the module were simplified and restricted (see Section Materials and Methods and Supplementary Material). It may be probable that the local interaction is influential for the persistence of neural activity over the next second as shown in BO-selective cells (O'Herron and von der Heydt, 2009). Whereas, these mechanisms such as feedback projections and local interactions within a cortical area may also play important roles in the neural coding of figure-ground segregation, these previous models did not discuss details of the time-course for the responses of BO-selective cells depending on the ambiguity of DOF. However, these models seemed to be difficult to reproduce the rapid transition of BO signals after representing the opposite DOF as reported by the physiological experiment (O'Herron and von der Heydt, 2009). Under such stimulus presentation, these two mechanisms might lead to a long latency in the transition of BO signals. Feedback projections to BO-selective cells appear to become effective after the establishment of the representation of the new object in a higher visual area. Local interactions within a cortical area seem to be activated after the activation of V2 neurons that receive the feedforward and/or feedback signals. In this mechanism, the selectivity of BO is developed via some steps of the interactions. In either case, it is not straightforward to design the figure-ground-cue-dependent and attention-dependent time-course in the neural dynamics. Further studies are necessary for understanding the neural mechanism of BO-selective cells.

Our model did not include the six-layered network of excitatory pyramidal neurons and inhibitory interneurons, which present a functional unit of cortical information processing. In particular, recent physiological experiments have shown that complex interactions between feedforward and feedback projections within cortical laminar structures result in figure-ground modulation (Self et al., 2013; van Kerkoerle et al., 2014). Several layered cortical models have been proposed to clarify the visual mechanism. Grossberg (2016) proposed a computational model for the cortical laminar structure to investigate how 2D surfaces are developed to 3D scene perception. Large-scale simulation is a powerful modern method for studying the detailed neuronal networks. Potjans and Diesmann (2014) have described a large-scale network of multi-layered cortical microcircuits, which was based on the integrated connectivity map derived from anatomical and electrophysiological data. Extending the Potjans' model, Wagatsuma et al. (2011, 2013b) explored the intra- and inter-laminar information flow during visual processings and attentional modulation. These works provided cues to help understand how feedforward and feedback signals were integrated within or between layered cortical microcircuits. The microcircuit network in the visual cortex might also play a key role in the induction of characteristic time-courses in BO-selective cells depending on figure-ground cues.

Simulation results of our network model predicted that feedforward signals via the surrounding suppression/facilitation, as well as the cortical interactions between early and parietal visual areas, play important roles in the time-course of BO-selective cells in intermediate-level vision, which may partially explain both the credibility of figure-ground segregation and detection of visual targets. Furthermore, spatial attention in early vision may modulate, at least partly, the activation speed of BO-selective cells and DOF perception response time. It is possible to examine these hypotheses from both psychophysical and physiological viewpoints. Our model simulations suggest that feedforward processing and cortical interactions play, at least in part, roles in the dynamics of BO-selective cells. These suggestions would lead to further understanding of visual mechanisms including object perception. Our results provide essential predictions related to the fundamental problems of figure-ground segregation and attentional selection.

## Author contributions

NW: design of the work, implementing the model, model simulations, analysis, drafting the work, and interpretation of data for the work. KS: design of the work, revising the draft of the work, analysis and interpretation of data for the work.

## Funding

This work was partly supported by KAKENHI Grant 26880019, 26280047, Research Institute for Science and Technology of Tokyo Denki University Grant Number Q16J-04, and RIEC Tohoku University Grant Number H28A13.

### Conflict of interest statement

The authors declare that the research was conducted in the absence of any commercial or financial relationships that could be construed as a potential conflict of interest.
