# Supplementary material for: Modeling the Time-Course of Responses for the Border Ownership Selectivity Based on the Integration of Feedforward Signals and Visual Cortical Interactions
Source: Front Psychol. 2017 Jan 20;7:2084. doi: 10.3389/fpsyg.2016.02084 (PMC5247462; doi:10.3389/fpsyg.2016.02084)
Supplement: Supplementary file 1 [file DataSheet1.pdf]

Supplementary Material

Modeling the Time-Course of Responses for the Border Ownership Selectivity Based on the Integration of Feedforward Signals and Visual Cortical Interactions

Nobuhiko Wagatsuma*, Ko Sakai

*** Correspondence:** Nobuhiko Wagatsuma: nwagatsuma@rd.dendai.ac.jp

1 Supplementary Materials for Detailed Descriptions of the Proposed Model

The model V1 cell for extracting the contrast from visual inputs and attentional modulation

A detailed mathematical description of the model V1 cell is given here. The input image, *Input*, is a 124x124 pixel, grey scale image with an intensity value ranging between zero and one. We designed the model so that 25 pixel corresponds to one degree (^{o}) in visual angle. Local contrast, $C_{\theta\omega xy}$, is extracted by the convolution of the image with a Gabor filter, $G_{\theta\omega}$;

$$C_{\theta\omega xy} = \sum_{i=1}^{m}\sum_{j=1}^{m} G_{\theta\omega}(i,j) Input(x-\frac{m}{2}+i, y-\frac{m}{2}+j) \tag{S1}$$

, where index x and y indicate spatial location, and ω indicates the spatial frequency. The local contrasts, $C_{\theta\omega xy}$, have the magnitude ranging between zero and two. Because we have limited input stimuli, we chose a single frequency of 0.5 ^{o} wavelength that is optimal for the extraction of contours from the stimuli. Orientation, θ, is chosen from 0, 90, 180 and 270 degree. m represents the number of pixels in the Gabor filter $G_{\theta\omega}$.

Spatial attention that is represented in PP modulates the contrast gain in V1 as in Equation 3 in the main text. Local contrast, $C_{\theta\omega xy}$, is modulated by attention that is given by the feedback from PP to V1, I_{xy}^{V1-PP}. The modulated contrast, $I_{\theta\omega xy}^{V1,exc}$, is given by the following equations, as proposed by (Lee et al., 1999; Peters et al., 2005):

$$I_{\theta\omega xy}^{V1,exc}(t) = \frac{(C_{\theta\omega xy})^{\gamma I_{xy}^{V1-PP}(t)}}{S^{\delta I_{xy}^{V1-PP}(t)} + \sum_{\theta\omega}\left(\frac{1}{N_i N_j}\sum_{i=1}^{N_i}\sum_{j=1}^{N_j} C_{\theta\omega(x-\frac{N_i}{2}+i)(y-\frac{N_j}{2}+j)}\right)^{\delta I_{xy}^{V1-PP}(t)}}, \tag{S2}$$

$$I_{xy}^{V1-PP}(t) = \chi \sum_{i=1}^{k}\sum_{j=1}^{l} W_{ij} F(A^{PP}_{(x-\frac{k}{2}+i)(y-\frac{l}{2}+j)}(t)), \tag{S3}$$

$$W_{ij} = \alpha \exp\left(-\frac{(i-\frac{k}{2})^2 + (j-\frac{l}{2})^2}{2\sigma_w{}^2} \right) \tag{S4}$$

, where W_{ij} represents connection weights of the Gaussian (Deco and Lee, 2004) with the standard deviation of σ_w (0.53 °). W_{ij} were chosen so that the total sum is one. $F(A(t))$ in Equation S3 is identical to that in Equation 2 in the main text. A_{xy}^{PP} shows the activity of a model PP cell as shown in Equation 8 in the main text. k and l show the spatial extent of the feedback from PP cells to a single V1 cell. α, χ, γ and δ are constants. S is a semi-saturation constant which prevents the denominator to be zero (Lee et al., 1999; Peters et al., 2005). In our simulation, we used $\alpha = 0.25$, $\chi = 0.6$, $\gamma = 4.0$, $\delta = 3.0$ and $S = 2.05$. These constants were chosen following the references (Deco and Lee, 2004; Lee et al., 1999; Peters et al., 2005). All constants were fixed throughout all simulations. The semisaturation constant S had an important role for preventing the magnitude of the modulated feedforward inputs $I_{\theta\omega xy}^{V1,exc}$ from being infinitely activated. Furthermore, this constant prevented the denominator to be zero. Major results were insensitive to the change of these parameters at least in the range between 75% and 150% of those used in the simulation (Wagatsuma et al., 2008; Wagatsuma et al., 2013). The denominator of Equation S2 shows inhibitory effects in V1. N_i and N_j represent the spatial range of the inhibitory effects, and the feedback from PP, I_{xy}^{V1-PP}, modulates this inhibition. N_i and N_j were set to 1.0°. The denominator of Eq.S2 functioned as the inhibitory unit in the V1 module. Spatial attention increases the contrast gain, thus the extracted signal at the attended location is enhanced.

The activity of a model V1 cell, $A_{\theta\omega xy}^{V1}$, is given by:

$$\tau \frac{\partial A_{\theta\omega xy}^{V1}(t)}{\partial t} = -A_{\theta\omega xy}^{V1}(t) + \mu F(A_{\theta\omega xy}^{V1}(t)) + I_{\theta\omega xy}^{V1,exc}(t) + I_{noise}^{V1}(t) \tag{S5}$$

, where I_{noise}^{V1} represents random noise and μ represents a scaling constant. In this simulation, we used $\mu = 0.95$. Equation S5 includes the contrast signal, $I_{\theta\omega xy}^{V1,exc}$, that is modulated by spatial attention so that the activities of model V1 cells at and around the attended location are increased.

The mechanism of the surrounding suppression/facilitation of a model BO-selective cell in V2

A mathematical description of the surrounding suppression/facilitation of a model BO-selective cell in V2 module is given here. The model determines BO based on surrounding contrast (Sakai and Nishimura, 2006; Sakai et al., 2012).

First, V2 module pools the contrast signals that are modulated by spatial attention in V1 module over space and frequency,

$$O_{\theta xy}^{1} = \sum_{\omega} \sum_{i=1}^{m} \sum_{j=1}^{m} W_{xyij} F(A_{\theta\omega(x-\frac{m}{2}+i)(y-\frac{m}{2}+j)}^{V1}), \tag{S6}$$

$$O^{1,cross}_{xy} = O^{1}_{(\theta+90)xy} + O^{1}_{(\theta+270)xy}, \quad \text{(S7)}$$

$$O^{1,iso}_{xy} = O^{1}_{\theta xy} + O^{1}_{(\theta+180)xy}. \quad \text{(S8)}$$

x and y represent the location of the classical receptive field (CRF) of the model cell. m indicates the spatial extent of feed-forward from V1 module. $O^{1}_{\theta xy}$ shows the feed-forward input from V1. Index *cross* and *iso* represent contrasts orthogonal and parallel to the preferred orientation, θ, of the cell, respectively. W_{ij} represents the Gaussian function as shown in Equation S4. $O^{1,cross}_{xy}$ and $O^{1,iso}_{xy}$ show the modulated contrast of cross-orientations and iso-orientations, respectively.

Second, the surrounding signal, $O^{2,BO}_{xyN}$, is given by a linear combination of contrast signals from excitatory and inhibitory regions that are defined by Gaussian functions as illustrated in Figure 1(B) in the main text.

$$O^{2,BO}_{xyN} = CF^{BO}_{xyN} - CS^{BO}_{xyN}, \quad \text{(S9)}$$

$$CF^{BO}_{xyN} = c_a \sum_{i=1}^{n_x} \sum_{j=1}^{n_y} F^{BO}_{N}(i,j) O^{1,cross}_{(x-\frac{n_x}{2}+i)(y-\frac{n_y}{2}+j)} \quad \text{(S10)}$$

$$CS^{BO}_{xyN} = c_b \sum_{i=1}^{n_x} \sum_{j=1}^{n_y} S^{BO}_{N}(i,j) O^{1,iso}_{(x-\frac{n_x}{2}+i)(y-\frac{n_y}{2}+j)}, \quad \text{(S11)}$$

CF^{BO}_{xyN} and CS^{BO}_{xyN} represent the contrast signals within the facilitatory and suppressive regions, respectively. The index N represents the type of model BO cells that are distinguished by their surround regions. We implemented 10 types of surround regions from a pool of Gaussians generated randomly to reproduce a diversity of BO selectivity (Zhou et al., 2000). F^{BO}_{N} and S^{BO}_{N} represent the facilitatory and suppressive regions of the model BO-selective cell. n_x and n_y indicate the spatial extent of facilitatory and suppressive regions. The combination of facilitatory and suppressive regions determines the property of BO selectivity. Such localized, asymmetric, and orientation dependent organization is observed in surrounding modulation in V1 neurons (Jones et al., 2002). c_a and c_b are connection strength. These constants for surrounding modulation (n_x, n_y, c_a and c_b) were determined following the references (Sakai and Nishimura, 2006). The balance of the facilitation and suppression determines modulation of the model BO-selective cell.

Third, the response of a model BO cell, $I^{V2-V1,BO}_{xyN}$, is determined based on a linear summation of the CRF signal, $O^{1}_{\theta xy}$, and the surround signal, $O^{2,BO}_{xyN}$.

$$\text{If } O^{1}_{\theta xy}(t) + O^{2,BO}_{xyN}(t) > 0 \quad \text{(S12)}$$

$$I^{V2-V1,BO}_{xyN}(t) = O^{1}_{\theta xy}(t) \times (O^{1}_{\theta xy}(t) + O^{2,BO}_{xyN}(t)), \quad \text{(S13)}$$

otherwise

$$I_{xyN}^{V2-V1,BO}(t) = 0 \tag{S14}$$

For the determination of direction of figure, the activities of model BO-selective cells are pooled to represent the population activities. For computing the BO signals, we took the summation of all activities of BO-right cells and that of BO-left cells (see the main text for details).

2 Supplementary Figure

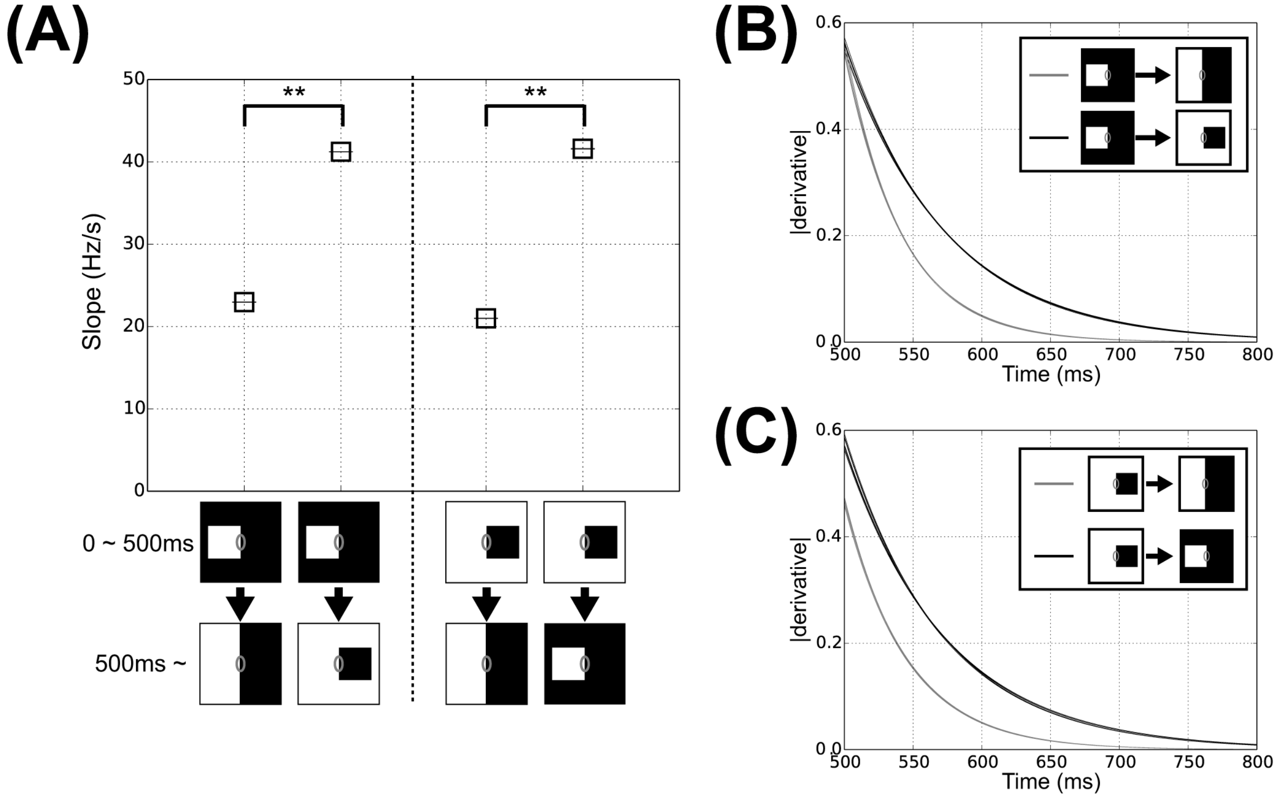

Figure S1. We fitted functions to BO signal *v* of each simulation trial after the replacement (>500 ms). We computed the slopes and time constants of BO signals *v* through fitting with exponential curves ($N(t) = N_0 \exp(-t/\tau)$, N_0 and τ meant the slope and time constant, respectively). Asterisks indicate significant differences between the stimulus sets (*t*-test: ** $p < 0.01$; * $p < 0.05$). **(A)** Slopes of BO signal *v* in our model. **(B)**, **(C)** Absolute values of $N(t)/\tau$ for each simulation trial, which corresponded to the speed of BO signals *v* in our model. Gray and black lines indicated absolute values of $N(t)/\tau$ for stimulus set shown in Figure 2(A) and (B), respectively.

References

Deco G, Lee TS. The role of early visual cortex in visual integration: a neural model of recurrent interaction. Eur J Neurosci. 2004; 20: 1089-1100.

Jones HE, Wang W, Sillito AM. Spatial organization and magnitude of orientation contrast interactions in primate V1. J Neurophysiol,

Lee DK, Itti L, Koch C, Braun J. Attention activates winner-take-all competition among visual filters. Nat Neurosci, 1999; 2: 375–381.

O’Herron, P., and von der Heydt, R. (2009). Short-term memory for figure-ground organization in the visual cortex. *Neuron.* 61, 801-809.

Peters RJ, Iyer A, Itti L, Koch C. Components of bottom-up gaze allocation in natural images. Vis Res, 2005; 45: 2397–2416.

Sakai K, Nishimura H. Surrounding suppression and facilitation in the determination of border ownership. J Cogn Neurosci, 2006; 18: 562-579.

Sakai K, Nishimura H, Shimizu R, Kondo K. Consistent and robust determination of border ownership based on asymmetric surrounding contrast. Neural Netw, 2012; 33: 257-274.

Wagatsuma N, Oki M, Sakai K. Feature-based attention in early vision for the modulation of figure-ground segregation. Front Psychol, 2013; 4: 123. doi:10.3389/fpsyg.2013.00123.

Wagatsuma N, Shimizu R, Sakai K. Spatial attention in early vision for the perception of border ownership. J Vis, 2008; 8: 22. doi: 10.1167/8.7.22.

Zhou H, Friedman HS, von der Heydt R. Coding of border ownership in monkey visual cortex. J Neurosci, 2000; 20: 6594-6611.
